# Digitally dedicated nurses: a nationwide cross-sectional study of associated career and digital factors in the workplace

**DOI:** 10.1186/s12913-025-13333-0

**Published:** 2025-10-15

**Authors:** Lotta Virtanen, Emma Kainiemi, Anu-Marja Kaihlanen, Tuulikki Vehko, Tarja Heponiemi

**Affiliations:** Department of Public Health and Welfare, Finnish Institute for Health and Welfare, P.O. Box 30, Helsinki, FI-00271 Finland

**Keywords:** Digital health, Telemedicine, Health information systems, Nursing, Work engagement

## Abstract

**Background:**

Nurses need job dedication to manage demanding working conditions, deliver high-quality care, and sustain professional interest. This dedication expands into digital dedication with electronic health records (EHRs) and other health information systems (HISs) prevailing in the nursing working day, combined with technologies for remote client interactions. This study examined (1) nurses’ digital dedication, (2) its variation based on career stage, position, EHR training opportunities, supportive HISs, and technology in use at work, and (3) whether EHR training opportunities moderate the association between career stage and digital dedication.

**Methods:**

A nationwide cross-sectional survey for registered nurses (*N* = 2926) was conducted in spring 2023 in Finland. Digital dedication was measured by perceived enthusiasm, inspiration, and pride in using nursing technologies. Associations of independent variables with dedication were analysed using linear regression, adjusted for background factors.

**Results:**

The mean digital dedication score was 2.91 (SD = 1.10, scale 1–5), indicating occasional dedication. In the multivariable model, late career stage was associated with lower dedication compared to early career stage (*b* = − 0.46, 95% CI [− 0.63, − 0.29]). EHR training opportunities moderated this association, weakening the negative effect of late career stage for those with training (*b* = 0.31, 95% CI [0.01, 0.62]). Additionally, nurse managers (*b* = 0.39, 95% CI [0.25, 0.52]), those using multiple HISs (*b* = 0.21, 95% CI [0.13, 0.30]), and working digitally with clients (*b* = 0.31, 95% CI [0.21, 0.40]) had higher scores compared to their counterparts. A stronger agreement with supportive HISs was associated with higher scores (*b* = 0.23, 95% CI [0.18, 0.28]).

**Conclusions:**

Digital dedication among nurses in Finland appears to be modest. Ensuring ongoing EHR training that addresses late career nurses’ needs is crucial; it can elevate their dedication to early career levels. Regular team discussions on technology use could enable managers to foster greater staff dedication. When invested in systems that genuinely support tasks, greater technology use can also improve dedication. Promoting digital dedication in nursing is important, as it could enable new roles in digital and remote work, particularly benefitting those for whom traditional roles have become too physically demanding.

**Supplementary Information:**

The online version contains supplementary material available at 10.1186/s12913-025-13333-0.

## Introduction

As a component of work engagement, dedication reflects a sense of enthusiasm, significance, and pride in one’s professional role [1]. These feelings are particularly essential for building resilience against the emotional, cognitive, and physical demands inherent in nursing. When nurses are dedicated to their work, they tend to demonstrate stronger organisational commitment and perform more effectively, which can contribute to higher quality of care [2–4]. Conversely, a lack of dedication is associated with intentions to leave the profession and actual turnover [2–4].

The integration of digital health technologies (DHTs) is transforming professional practices and the working environment in nursing, and may change perceptions of the profession. DHTs for digital client work include secure messaging, teleconsultations (via video, audio, or chat), and digital care pathways, enabling nurses to assess and monitor health conditions, provide care instructions, and communicate with clients either in real-time or asynchronously [5, 6]. This also facilitates nurses’ remote working. Long-established health information systems (HISs), such as electronic health records (EHRs) and ancillary systems, remain essential for supporting daily traditional and digital client work, as well as administrative processes [7, 8]. In the future, DHTs will increasingly be complemented by functionalities based on artificial intelligence [9]. These changes could reduce workload, create new tasks and career opportunities, and offer flexible working arrangements [5, 10, 11], potentially making nursing more attractive and supporting continued employment until or beyond the official retirement age [12–14]. Such benefits may be limited if nurses are not dedicated to using technologies in their work.

Several reviews have synthesised nurses’ attitudes and satisfaction with DHTs, which indicate that nurses can be receptive to technology use and perceive benefits for nursing tasks, information management, and job control [5]. However, studies have also reported negative impacts, including usability challenges that complicate tasks, the introduction of additional duties on top of existing responsibilities, and reduced caregiving time [5, 15–19]. What is lacking is an understanding of nurses’ dedication to using DHTs, which would provide a more nuanced perspective of their professional engagement. Unlike general attitudes or satisfaction, which describe readiness for, contentment with, or the absence of hassles related to technologies, digital dedication reflects how nurses emotionally engage with and commit to using DHTs in their daily practice. This conceptualisation applies the definitional distinction between general job satisfaction and job dedication [1, 20], and extends the original concept of techno dedication introduced in research on teachers [21] to the use of technology in nursing. As technologies become increasingly prevalent also in nursing, often as mandatory tools, dedication to their use can be viewed as an essential professional sentiment alongside sufficient competence. It can also positively affect clients’ use of digital services, as nurses dedicated to using DHTs have been found to be more likely to recommend these technologies to their clients compared to their less dedicated colleagues [22].

Existing literature has identified some potential career-related factors that could be associated with digital dedication. Closely linked to digital dedication is technology acceptance, which may be more readily experienced in early career stages compared to later ones [23, 24]. One reason for this is that technological changes can provoke resistance by disrupting established work routines. For example, EHRs have standardised documentation, which may have been difficult to adopt for nurses who have long been accustomed to different practices in documenting client information [23, 24]. However, early career nurses can face distinct challenges: adapting to digital client work while still mastering traditional care can be burdensome, as research on doctors suggests [25]. Despite assumptions that younger generations are more adept with technologies, nurses entering the workforce have also expressed concerns that DHTs could depersonalise care and contradict their career choice motivations [26]. Furthermore, position within the career, whether as a staff nurse or nurse manager with client duties, might also shape commitment to new innovations, as those with managerial responsibilities have reported fewer negative effects related to digitalisation compared to frontline staff [16].

Moreover, organisational factors related to digital tasks may contribute to digital dedication. Work wellbeing theories posit that adequate training opportunities can facilitate the achievement of work goals and foster professional development [1, 27]. Nurses have perceived these opportunities to increase their commitment to work [28, 29]. In the context of digital tasks, competence should be regularly upgraded for EHRs due to updates with new functionalities, usability improvements, and changes in maintaining data security, which alter the systems’ functioning and use. As EHR use takes up a considerable portion of working time [18, 19] and is central to both traditional and digital client work, it is reasonable to assume that high-quality training in EHRs could improve digital dedication. Additionally, if the DHTs procured by the workplace are designed to support nursing, this may encourage their adoption [30]. Nurses who have the opportunity to use these technologies in their work are likely to become familiar with the functions, which, in turn, might strengthen commitment to their utilisation.

This study aimed to assess digital dedication, a novel measure [21] of the level of enthusiasm, significance, and pride in using DHTs in nursing, among registered nurses in Finland. Identifying factors associated with digital dedication is essential to promoting meaningful nursing practice and the effective use of digital investments in health systems. Thus, potential variations in digital dedication were examined based on career and digital factors in the workplace, including training opportunities for EHR use, supportive HISs, and current use of technology in client work. Additionally, the moderating effect of training opportunities for EHR use on the potential association between career stage and digital dedication was assessed. The hypothesis was that the opportunity to receive training in the use of evolving EHRs, which all nurses utilise throughout the working day, could improve overall digital dedication differently across career stages. This assumption was based on the premise that training could address the challenges related to changes in documentation practices with EHRs, which may appear differently depending on career length [23, 24]. The following research questions were formulated:

1. What is the level of digital dedication among registered nurses in Finland?
2. Are nurses’ career stage, position, training opportunities for EHR use, supportive HISs, the number of HISs used in client work, and working digitally with clients associated with digital dedication?
3. Do training opportunities for EHR use moderate the potential association between career stage and digital dedication?

## Methods

### Study design

A cross-sectional study of the data collected for the national initiative on *Monitoring Digital Healthcare and Social Welfare *was conducted to assess the current stage of digitalisation in Finland [31]. The Strengthening the Reporting of Observational Studies in Epidemiology guidelines [32] were adhered to in the reporting of this study. 

### Setting

This study was conducted in Finland, where the education of registered nurses provides the baseline for public health nurses, midwives, and paramedics [33]. The majority of nurses are employed in the public sector, where wellbeing services counties are responsible for organising health and social care services, while approximately one-fifth work in the private sector [34]. Finland ranks among the foremost nations in terms of digitalisation [35]. Although the usage intensity of most HISs is exceptionally high, not all systems integrate seamlessly, resulting in the concurrent use of multiple systems [36]. For example, many counties share a common core EHR system, but discipline-specific ancillary systems can be partially incompatible across different care sectors, organisations, and units [8, 36]. Since nurses can work across health and social care settings, the systems they use vary depending on the setting. In this study, the term HISs also encompasses systems used in social care, and EHRs can refer to client information systems as well.

Nurses hold a central position in promoting digitalisation, as they represent a large professional group that often serves as the primary point of contact for clients [37]. They can integrate DHTs into care paths for suitable clients, which is essential for promoting technology adoption and reducing digital exclusion among clients who do not independently access these technologies [38–40]. Nurses also perform a significant amount of digital client work, including secure messaging, teleconsultations, and digital promotion of care, with secure messaging being the most frequently performed method, often on a daily or weekly basis [6]. Nationwide digital platforms, such as a patient data repository, digital self-care programmes, and digital symptom checkers, support this work. Additionally, many wellbeing services counties utilise digital clinics, enabling nurses to encounter clients remotely [37].

### Data collection and participants

An online *Survey on information systems for registered nurses in 2023* was conducted over a two-week period in April 2023. The survey primarily addressed nurses’ user experiences of EHRs, proficiency in using the systems and perceived benefits, but also included questions regarding nurses’ engagement with DHTs. The survey was developed in multidisciplinary collaboration with national stakeholders and educational institutions, with its origins rooted in previous national data collections involving firstly doctors [41, 42], and tailored later for registered nurses [43].

An invitation to participate, including the link to the survey, was disseminated via email to working-aged (aged 18–65) registered nurses by two associations, ‘Tehy’– the Union of Health and Social Care Professionals, and the Finnish Nurses Association. To enhance the response rate, the survey was advertised in advance through the communication channels of the Finnish Institute for Health and Welfare and the associations. Additionally, a reminder to respond was sent to registered nurses during the data collection period. Nurses were able to respond in Finnish, Swedish, or English.

Eligibility for the survey required that participants were registered nurses who used HISs in their current role. The survey focused on these systems and therefore, responding was automatically closed to those who indicated that they do not use them in their work, such as those who have transitioned to other professions. For this study, an additional inclusion criterion was applied: staff nurses and nurse managers were eligible if they used HISs in client work, despite having managerial responsibilities. This criterion aligned with the study’s focus on digital dedication among those whose roles involved direct client duties to at least some extent.

### Variables

Additional file 1 presents a detailed description of the study variables.

#### Dependent variable

*Digital dedication* was measured using one of the three dimensions belonging to the TechnoWES instrument, which was developed by Mäkiniemi et al. [21]. This dimension was evaluated to be most suitable for the nursing work context, and it assessed how often (1 = not at all – 5 = daily) the following kinds of feelings and thoughts arise: 1) I am enthusiastic about utilising technology in my job (*enthusiasm*); 2) Utilising technology inspires me in my job (*significance*); and 3) I am proud that I utilise technology in my work (*pride*). A composite mean score was calculated from the items, with a higher score indicating a greater level of dedication, and internal consistency was assessed using Cronbach’s alpha (α). The measurement has previously been used and validated among teachers in Finland [21].

To apply the items in this study, nurses were instructed to consider the emotions related to the DHTs used in their work, such as EHRs, end-user devices, and other technologies used in clinical care and teleconsultations. Before disseminating the survey, the items were piloted among nurses studying for an additional degree in information management who also considered the items to be understandable and relevant in nursing. Digital dedication has been used as an independent variable in previous nursing studies where a higher score in digital dedication has been associated with working digitally with clients [6] and referring clients to use DHTs [22].

#### Independent variables

Two variables described the respondents’ careers: *Career stage* was determined based on the time since completion of the degree in nursing and age of the respondents: 1 = early career (graduated within the last 5 years), 2 = mid-career, 3 = late career (approaching retirement, aged 60 or over); *Position* was coded as 1 = staff nurse, 2 = nurse managers with client duties.

Four variables outlined digital factors in the workplace. Most of them were linked to HISs that provide the foundation for digital client work. *Training opportunities for EHR use *was based on respondents’ opinion (recoded as 1 = disagree–neutral, 2 = agree) on the following statement: ‘Employer offers continuing training in electronic health records or client information systems use’. *Supportive HISs *was measured from opinions (1 = fully disagree – 5= fully agree) regarding six statements on how HISs procured in the workplace support the carrying out of duties, such as ‘Information systems help to avoid duplicate tests and examinations’ and ‘Information systems help to ensure continuity of care’, which have been used in previous studies among nurses [43, 44]. A composite mean score was calculated from the items (α = 0.85), with a higher score indicating greater perceived support. The *number of HISs used in client work* was based on how many systems respondents log in to in their work with clients (recoded as 1 = 1–2 systems, 2 = 3 or more). *Digital client work *wasbased on whether the respondents’ work involves digital contacts with clients, excluding telephone work (1 = no, 2 = yes).

#### Background variables

Respondents were also asked about their background in terms of *gender*, *workplace*, *location of employment*,* digital work skills*, and experienced *stress related to HISs*. *Age* was only used for descriptive statistics due to its high correlation with career stage. Additionally, *professional qualification* was used to describe the sample.

### Data analysis

**Fig. 1:**
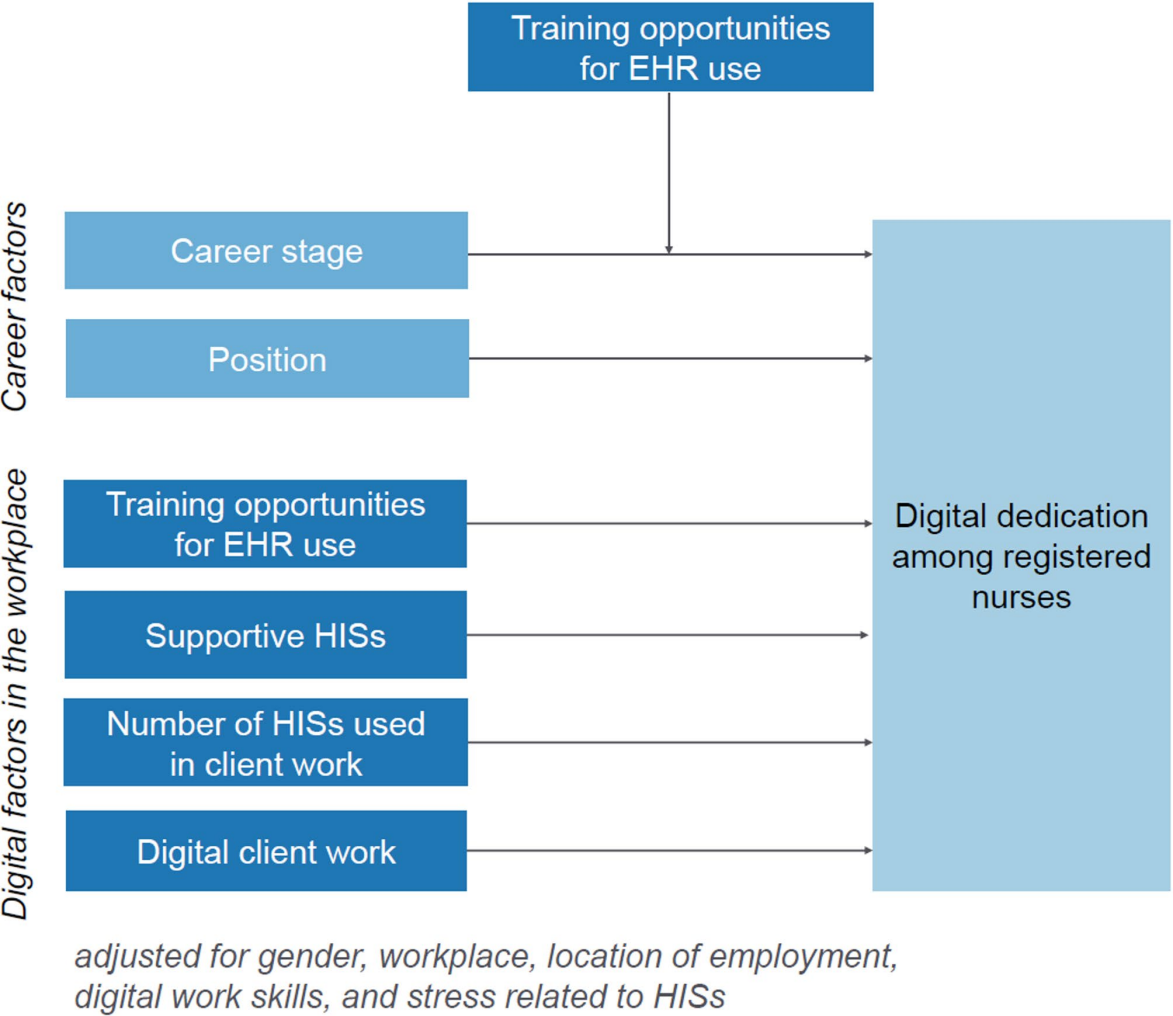
Final model to be tested in the study. *Note. EHR* Electronic health record, *HISs* Health information systems

Descriptive statistics were used to describe the respondents and their levels of digital dedication. For the linear regression analysis, missing data was excluded. Linear regressions were conducted in three steps to analyse the associations of the independent variables (career and digital factors in the workplace) with digital dedication. Initially, separate univariable linear regression models were performed, wherein the effect of each independent variable on the digital dedication was assessed individually. The analysis continued with multivariable linear regression to assess whether these associations would change when all independent variables were simultaneously added to the multivariable model 1. This multivariable model was adjusted for gender, workplace, digital work skills, and stress related to HISs, because preliminary analyses (ANOVA and chi-square tests) indicated that these factors could introduce variation in the distribution of the dependent and independent variables. Additionally, the location of employment (wellbeing service county) was adjusted due to the unequal representation of respondents across the wellbeing services counties of Finland. Finally, a multivariable model 2 was created for which the interaction term of career stage and training opportunities for EHR use was introduced, in addition to including the same independent variables and adjustments as in multivariable model 1 (Fig. 1). Assumptions for linear regression, such as the absence of multicollinearity [45], were examined. The analyses were conducted using the lm() function in R (version 4.2.1) [46].

## Results

A total of 2970 registered nurses who used HISs in their work participated in the survey. After excluding 44 respondents who used HISs solely for administrative tasks, the final sample comprised 2926 participants. The majority were registered nurses without additional qualifications (*n* = 2355, 81%), while a smaller proportion were additionally qualified as public health nurses (*n* = 325, 11%), midwives (*n* = 150, 5%), paramedics (*n* = 69, 2%), or held other nursing qualifications (*n* = 27, 1%), such as specialist qualifications or those based on earlier educational structures. Most respondents (*n* = 2047/2922, 70%) were identified as mid-career nurses, while 16% (*n* = 462) were in their early career and 14% (*n* = 413) in their late career stage. Slightly under a tenth (*n* = 273/2926) were nurse managers with client duties. Different workplaces were represented, but most often nurses worked in outpatient (*n* = 853/2924, 29%) or inpatient (*n* = 667, 23%) care. Approximately a third agreed that their employer offered opportunities for continuing training for EHR use (*n* = 884/2915). The mean score of supportive HISs was 3.16 (SD = 0.86), suggesting that respondents slightly agreed with statements about the support that these systems provide for nursing duties. Approximately a third regularly used three or more HISs in client work (*n* = 975/2926) and worked digitally with clients (*n* = 861/2895). Table 1 presents a detailed overview of the characteristics of the participating registered nurses and the variables included in the study.

**Table 1:** Characteristics of the studied registered nurses and study variables (*n* = 2926^a^)

Characteristic			Value
***Background***			
Age			*n* = 2922, *n* (%)
< 35			454 (15.5)
35–44			689 (23.6)
45–54			912 (31.2)
55–65			867 (29.7)
Gender			*n* = 2926, *n* (%)
Woman			2687 (91.8)
Man			212 (7.3)
Other or prefer not to say			27 (0.9)
Workplace			*n* = 2924, *n* (%)
Acute care			503 (17.2)
Inpatient care			667 (22.8)
Outpatient care			853 (29.2)
Home-based care			197 (6.7)
Supportive housing and care facilities			316 (10.8)
Other			388 (13.3)
Digital work skills			*n* = 2867, *n* (%)
Tolerable–Satisfactory			468 (16.3)
Good–Excellent			2399 (83.7)
Stress related to HISs^b^			*n* = 2916, mean (SD)
			3.72 (1.17)
***Career factors***			
Career stage			*n* = 2922, *n* (%)
Early career			462 (15.8)
Mid-career			2047 (70.1)
Late career			413 (14.1)
Career position			*n* = 2926, *n* (%)
Staff nurse			2653 (90.7)
Nurse manager with client duties			273 (9.3)
***Digital factors in the workplace***			
Training opportunities for EHR use			*n* = 2915, *n* (%)
Disagree–Neutral			2031 (69.7)
Agree			884 (30.3)
Supportive HISs^c^			*n* = 2916, mean (SD)
			3.16 (0.86)
Number of HISs used in client work			*n* = 2926, *n* (%)
1–2			1951 (66.7)
3 or more			975 (33.3)
Digital client work			*n* = 2895,* n* (%)
No			2034 (70.3)
Yes			861 (29.7)

^a^Due to missing information in some variables, the number of respondents varies between 2915 and 2926, ^b^ scale 1–6, ^c^ scale 1–5. *EHR* Electronic health record, *HISs* Health information systems

### Digital dedication

**Fig. 2:**
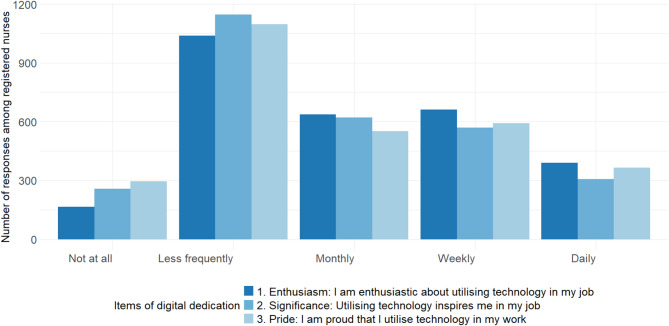
Responses to the three items regarding digital dedication among registered nurses (*n* = 2893–2901)

The responses describing the respondents’ DHT-related enthusiasm, significance, and pride were consistent, supporting their combination into a digital dedication composite score (α = 0.92) (Fig. 2). The mean digital dedication among the respondents was 2.91 (SD = 1.10, *n* = 2901) on a scale ranging from 1 (not at all) to 5 (daily). There was considerable variability in the levels of digital dedication among respondents in different career stages: the mean score was lowest among late career nurses (m = 2.67, SD = 1.07, *n* = 408) and highest among early career nurses (m = 3.07, SD = 1.10, *n* = 459), whereas mid-career nurses fell in between (m = 2.92, SD = 1.10, *n* = 2030).

### Variables associated with digital dedication

**Fig. 3:**
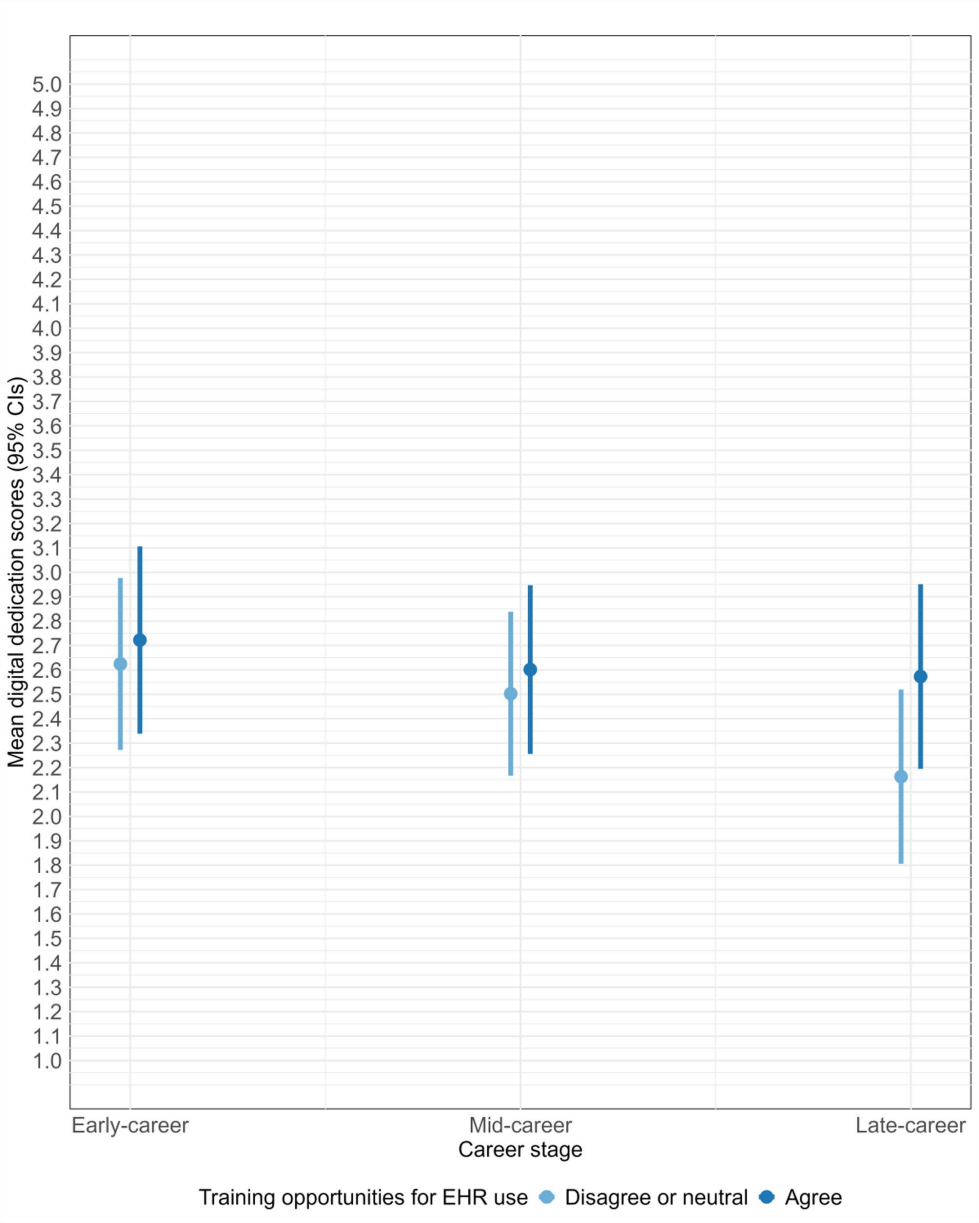
The moderating effect of training opportunities for EHR on digital dedication among registered nurses. *Note.* The figure illustrates the interaction between career stage and training opportunities for EHR use on the mean digital dedication scores, with 95% CIs, among registered nurses (*n* = 2777). The means are adjusted for the other variables in multivariable model 2, and the values represent unstandardised predicted scores

In multivariable linear regression model 1, after adjusting for other variables, mid-career (*b* = − 0.12, 95% CI [− 0.23, − 0.02]) and late career nurses (*b* = − 0.36, 95% CI [− 0.50, − 0.21]) had lower digital dedication scores compared to early career nurses (Table 2). Multivariable model 2 introduced the interaction term for career stage and training opportunities for EHR, of which late career stage (relative to early career) and agreeing with training opportunities for EHR (relative to disagreeing or being neutral) showed statistical significance (*b* = 0.31, 95% CI [0.01, 0.62]) (Table 2). In this model, the main effect of career stage reflects conditions where nurses either disagreed with or were neutral about training opportunities. Among nurses without training opportunities for EHR use, those at a late career stage had, on average, digital dedication scores 0.46 points (95% CI [− 0.63, − 0.29]) lower than those at early career stage. However, in the presence of training opportunities, the negative association between late career stage and digital dedication weakened. Late career nurses with training had, on average, digital dedication scores 0.15 points lower than early career nurses without training. This conditional effect is visually represented in Fig. 3.

**Table 2:** Linear regression models for digital dedication among registered nurses (*n* = 2777–2876)

Independent variable	Univariable models *n* = 2850–2876		Multivariable model 1 *n* = 2777		Multivariable model 2 with an interaction term *n* = 2777	
	*b*	95% CI	*b*	95% CI	*b*	95% CI
*Career factors*						
Career stage						
Early career	ref.		ref.		ref.	
Mid-career	**−0.147**	**−0.258, −0.036**	**−0.123**	**−0.231, −0.015**	−0.122	−0.248, 0.004
Late career	**−0.402**	**−0.548, −0.256**	**−0.355**	**−0.499, −0.212**	**−0.462**	**−0.634, **−**0.290**
Career position						
Staff nurse	ref.		ref.		ref.	
Nurse manager with client duties	**0.379**	**0.241, 0.516**	**0.382**	**0.245, 0.519**	**0.386**	**0.248, 0.523**
*Digital factors in the workplace*						
Training opportunities for EHR use						
Disagree–Neutral	ref.		ref.		ref.	
Agree	**0.316**	**0.230, 0.403**	**0.146**	**0.059, 0.233**	0.098	−0.004, 0.202
Supportive HISs	**0.293**	**0.249, 0.339**	**0.229**	**0.179, 0.278**	**0.227**	**0.178, 0.276**
Number of HISs used in client work						
1–2	ref.		ref.		ref.	
3 or more	**0.179**	**0.095, 0.264**	**0.216**	**0.131, 0.301**	**0.212**	**0.128, 0.298**
Digital client work						
No	ref.		ref.		ref.	
Yes	**0.344**	**0.257, 0.431**	**0.305**	**0.212, 0.397**	**0.305**	**0.212, 0.397**
*Interaction term*						
Career stage × Training opportunities for EHR use						
Early career × Disagree–Neutral					ref.	
Mid-career × Agree					0.001	−0.239, 0.241
Late career × Agree					**0.312**	**0.007, 0.618**

*b *Unstandardised beta coefficient indicates the average change in the digital dedication score (on a scale of 1–5) under the following conditions: a) in a specific variable group compared to a reference group (for categorical independent variables), or b) for a one-unit change in supportive HISs (a continuous independent variable). *CI* Confidence interval, *ref.* Reference, *EHR *Electronic health record, *HISs* Health information systems. The two multivariable models were adjusted for gender, workplace, location of employment, digital work skills, and stress related to HISs. Statistically significant values (*p* <.05) are highlighted in bold

Multivariable model 2, which included the interaction term, was significant, *F* (39, *n* = 2777) = 10.60, *P* <.001, with the independent variables collectively explaining 12% of the variance (adjusted R^2^) in digital dedication. No violations of the assumptions for linear regression were detected. When controlling for other variables in the model, nurse managers had higher digital dedication scores compared to staff nurses (*b* = 0.39, 95% CI [0.25, 0.52]). A 1-point increase in supportive HISs scores was associated with increased digital dedication scores (*b* = 0.23, 95% CI [0.18, 0.28]). Additionally, nurses who used three or more HISs in client work (*b* = 0.21, 95% CI [0.13, 0.30]) and those who worked digitally with clients (*b* = 0.31, 95% CI [0.21, 0.40]) had higher digital dedication scores compared to their counterparts who did not use multiple HISs or work digitally with clients. Additional File 2 presents the linear regression coefficients for all background variables used as adjustments in the multivariable models.

## Discussion

This study delved into nurses’ enthusiasm, significance, and pride related to the use of DHTs in nursing, alongside its potential associated career and digital factors in the workplace. The findings suggested that, on average, registered nurses in Finland experience digital dedication occasionally (i.e. at least nearly monthly), which seems modest given the increasing integration of DHTs into nursing practice. This dedication clearly decreased towards the end of their career, but employer provision of continuing training for EHR use moderated the association between the late career stage and lower levels of digital dedication. Thus, while EHR training is essential for skill development among nurses at all career stages, it may hold particular significance for late career nurses’ digital dedication. Additionally, nursing managers with client duties, those who used multiple HISs in client work, and those who worked digitally with clients had greater levels of digital dedication compared to their counterparts. A stronger agreement with the support of HISs for nursing was also associated with increased digital dedication.

The analysis revealed an association between career stage and digital dedication, with early career nurses having the highest average scores. This finding aligns with previous studies that indicate greater technology readiness among nurses with less work experience [30, 44, 47, 48]. This may be attributed to their familiarity with modern technology, the inclusion of DHT-focused training programmes in contemporary nursing education [49], and fewer established routines that would need to be altered for technology use [23, 24]. The results can also be interpreted in light of a study by Huber and Schubert [50], which revealed that the younger generations may value professional ambition in nursing more than older generations. Consequently, younger nurses might be particularly committed to the opportunities that DHTs present to the profession, such as specialisation in nursing informatics and the new roles it entails [10, 51]. While embracing digitalisation may increase the appeal of nursing and attract new professionals, it is essential that technology enriches rather than replaces the personalised care that often motivates individuals to pursue this career [26]. 

A key finding from this study is that late career nurses scored noticeably lower for digital dedication compared to their early career counterparts in the absence of ongoing EHR training. However, this negative relationship appears to be mitigated when employers provide such training. Another finding was that this training had only a minimal effect on mid-career nurses’ dedication, which was similar to that of early career nurses. Nevertheless, when compared to early career stage, the negative relationship between mid-career stage and digital dedication was less pronounced than the stronger negative relationship observed between late career stage and digital dedication. The findings support the hypothesis that training opportunities for EHR use can improve digital dedication differently across career stages. Specifically, the positive effect of EHR training on late career nurses’ dedication may be attributed to EHR being the first significant DHT, and the accompanying changes in documentation practices, which may have introduced resistance for these nurses with established work routines for documentation [23, 24]. EHR training that addresses negative sentiments related to the work changes and provides support for adaptation may therefore particularly increase late career nurses’ interest in newer nursing technologies as well.

While the improvement in digital dedication among late career nurses with EHR training was notable, further enhancements in training content may be necessary to more effectively reduce the career-stage disparity. Workplaces should recognise and accommodate older age-related needs, such as potentially longer learning times when planning training. Studies also call for more systematic organisation of continuing training in digital competence, noting that current practices vary substantially between and within European countries, can depend on individual employers’ resources, and often lack strategies involving empathy, encouragement, and user-centredness to enhance motivation towards using DHTs [51, 52]. The findings also suggest an area for future research: investigating whether improving lifelong continuing training in a broader range of DHTs could help to mitigate the digital divide among nurses at different career stages. The added value that training opportunities for technology use could have for digital dedication in later career is crucial, as digitalisation enables flexible tasks like remote work [6, 10, 11], potentially extending the careers for those unable to perform traditional nursing duties due to physical limitations. Reducing physical strain and providing better training opportunities have previously been found to increase late career nurses’ willingness to delay retirement [13, 53].

It was also found that nurse managers experienced greater levels of digital dedication compared to staff nurses, which aligns with a previous finding on the less negative perceptions of digitalisation’s effects on work among those in managerial roles [16]. One explanation might be the commitment to upper management decision-making that often comes from a managerial position. Although all nurse managers in this study also performed client work, it must be noted that digital dedication measured the feelings related to all technologies used at work. Hence, nurse managers’ more frequent digital dedication may also partly stem from the use of HISs for administrative tasks such as staff allocation, resource tracking, and information retrieval for knowledge management purposes [7], which may be perceived as more beneficial compared to the technologies used by staff nurses for clinical tasks. Nevertheless, the higher digital dedication scores among nurse managers enable them to act as role models, informing about the rationale for new implementations, and listening to and considering employees’ preferences during times of change. Such practices align with leadership models known to promote technology adoption and general work engagement [3, 30, 54]. As Cho et al. [55] aptly stated, fostering positivity is crucial, because hidden resistance behaviour often occurring with mandatory DHT use might lead to inappropriate technology use and compromise the quality of care.

Moreover, the results suggested that nurses who work digitally with clients can have greater levels of digital dedication compared to those who interact with clients only through traditional means. Interestingly, a previous study found that nurses who work more digitally with clients can experience higher levels of digital dedication than those working less digitally with clients [6]. These findings imply a bidirectional relationship: nurses who perceive digital dedication may seek out more roles enabling digital client work, while performing these roles may further enhance their dedication by making the benefits of DHTs more tangible. The potential to include digital tasks for a greater number of nursing professionals with the aim of strengthening digital dedication, however, often depends on the nature of tasks, the client groups encountered, and the technologies available at the workplace [6].

In addition to digital client work, the use of multiple HISs was associated with higher levels of digital dedication in this study. Although these findings could be interpreted to suggest that more digital tasks may also promote wellbeing at work, as overall job dedication has been linked to various aspects of occupational wellbeing [2–4], previous studies have indicated that conducting numerous teleconsultations and using multiple HISs can increase healthcare professionals’ psychological pressure at work [25, 56, 57]. The necessity to use multiple systems often arises from a lack of interoperability [36], requiring professionals to document the same client information in several different systems to ensure continuity of care [43, 57]. To mitigate the potential adverse effects of digital tasks, workplaces should plan sufficient time for documentation duties and various forms of client work [6, 25, 56]. This planning should ensure that designated periods are set for digital tasks and traditional clinical work, all in a manner that is meaningful for professionals and within the operational capabilities of the workplace.

### Limitations

The results are subject to certain limitations. The statistical modelling explained a relatively small proportion (12%) of the variance in digital dedication. Although limited explanatory power is typical for these types of studies [58], it may imply that other important associated factors not measured in this study could exist. For example, the analysis could not be adjusted for personal resources (e.g. psychological capital) or broader job features associated with work engagement [3, 4]. The survey did not investigate participation in continuing training for EHRs use, satisfaction with the training content, nor participation in training for other DHTs used in their work, which might be more strongly associated with digital dedication than the availability of EHR training opportunities measured in this study. Respondents’ subjective perceptions of digital dedication may have been influenced by their feelings towards specific technologies, such as irritation or enthusiasm, potentially biasing their overall assessment. As this was a cross-sectional study, causality cannot be inferred from the results. Moreover, the response rate could not be determined, as nurse associations distributed the survey. Nevertheless, the relatively large sample size and diverse backgrounds of the respondents suggest that the target group [59] is well represented. The exception was that nurses working in some areas in Finland were overrepresented, which was addressed by adjusting for location of employment in the analysis. Lastly, it is important to acknowledge that the generalisability of the findings to nurses’ experiences in other countries may be limited due to differences in health systems, cultural contexts, and levels of technological development.

### Conclusions with implications for workforce strategy and policy

This study suggests that registered nurses in Finland may feel a rather modest level of enthusiasm, significance, and pride in using DHTs, which is concerning given that such dedication can be crucial for the meaningfulness of their work. Dedication can vary based on career and digital factors in the workplace. For example, nurses with fewer years of work experience expressed the most digital dedication, providing initial insights into the potential of DHTs to be an inspiring part of work among future nursing professionals. However, efforts to promote digital dedication among currently practising nurses should be undertaken given that digitalisation will inevitably increase in health systems and can rapidly become more prominent in the work of nurses across various work settings.

With EHRs already prevailing in the nursing working day and these systems continuously evolving, every workplace must ensure the provision of training to update staff skills. Simultaneously, it is important to recognise that, as this research revealed, EHR training can be particularly beneficial in elevating the overall digital dedication of late career nurses to a level comparable to their colleagues in the early career stage. To reinforce the positive effects, workforce strategies should focus on developing EHR training programmes that could better address the needs of older age, such as allocating additional time for learning, emphasising practical learning over lecture formats, and appointing technologically experienced nurses to mentor their peers in a similar career stage [60, 61]. Importantly, these learning methods should be made available to all nurses alongside other training options, so that individuals, regardless of career stage, can choose the most suitable approach for them. This approach ensures that training is inclusive and prevents age-based discrimination in the workplace.

Additionally, the greater levels of digital dedication among nurse managers with client duties compared to staff nurses suggests a need to increase their interactions. Regular in-person team discussions about digital tools in client work would allow for addressing related concerns and sharing feedback in a positive atmosphere [62]. These discussions should be facilitated by managers who are engaged in client work and therefore understand the everyday realities of nursing, while also being committed to supporting digitalisation as part of the organisation’s strategic development.

It is also noteworthy that higher dedication was observed among nurses who were already working digitally with clients, using multiple systems in client work, and perceiving that the systems supported their work. These nurses should be supported in taking on mentoring or development roles related to DHTs, which may create new avenues for career growth in a field where progression opportunities have traditionally been limited. As digital tasks become increasingly standard, this development may, in itself, mitigate resistance and prejudices, provided that healthcare decision-makers ensure that the focus of new procurements is based on their genuine ability to enhance practical nursing work. Decisions should be grounded in scientific evidence on the effectiveness of DHTs, obtained through robust research designs and multidimensional indicators that, in addition to organisational efficiency, account for overall workflow, occupational wellbeing, and quality of care. From a workforce strategy perspective, data-driven management should be strengthened by regularly monitoring the effects of existing technologies on nurses’ work to inform both technological development and work organisation [63]. Offering meaningful technologies and fostering nurses’ active willingness to integrate them into their work may further contribute to improved job satisfaction and retention rates in nursing.

## Supplementary Information

Additional file 1. Study variables. The original questions, response options, and their transformation into the study variables

Additional file 2. Regression coefficients for background variables. Linear regression coefficients for background variables in models examining digital dedication among registered nurses

## Data Availability

The datasets used and analysed during the current study are not publicly available and cannot be shared due to data protection regulations and ethical considerations.

## Abbreviations

DHT: Digital health technology
EHR: Electronic health record
HIS: Health information system
