# Supplementary material for: Digitally dedicated nurses: a nationwide cross-sectional study of associated career and digital factors in the workplace
Source: BMC Health Serv Res. 2025 Oct 15;25:1365. doi: 10.1186/s12913-025-13333-0 (PMC12522463; doi:10.1186/s12913-025-13333-0)
Supplement: Supplementary file 1 — Additional file 1. Study variables. The original questions, response options, and their transformation into the study variables [file 12913_2025_13333_MOESM1_ESM.docx]

# **Additional file 1**

## **Study variables**

The *Survey on information systems for registered nurses in 2023* belongs to the national initiative on *Monitoring Digital Healthcare and Social Welfare* in Finland. The survey form has been published online [1]. Detailed information on the variables used in our study is provided in Table A1.

**Table A1**

*The original questions, response options, and their transformation into the study variables*

| Variable | Question(s) | Response options | Variable recoding/computation | Additional information |
| --- | --- | --- | --- | --- |
| **Dependent variable** | | | | |
| *Digital dedication* | The following question concerns your thoughts on the use of digital technology in work (e.g. electronic health records or client information system, terminal services, technologies used in patient care, technologies related to teleconsultations, digital interaction channels). How often do you have the following kinds of feelings and thoughts?   1. I am enthusiastic about utilising technology in my job. 2. Utilising technology inspires me in my job. 3. I am proud that I utilise technology in my work. | 1. Daily 2. Weekly 3. Monthly 4. Less frequently 5. Not at all | The response options were reverse coded. The three items were used as a composite mean score (Cronbach’s alpha [α] = 0.92). | The items belong to the techno dedication dimension of the TechnoWES instrument developed by Mäkiniemi et al. [2]. The instrument has previously been used and validated among teachers in Finland. Additionally, it has been used as an independent variable in previous nursing studies, associated with working digitally with clients [3] and referring clients to use DHTs [4]. |
| **Independent variables** | | | | |
| *Career stage* | Year of completion of the degree in nursing | 1970–2023 | Time since graduation and age were calculated from the year of data collection (2023), and used to compute the variable, coded as:  1 = Early career (graduated within the last 5 years)  2 = Mid-career, including respondents who had longer than 5 years from graduation and were not yet approaching retirement age  3 = Late career (age 60 years or over)  Two respondents, recently graduated but approaching retirement age, were categorised within the early career group, under the assumption that they would likely continue in their recently acquired career rather than opting for early retirement. | The same measure was used in a previous nursing study and was not found to be associated with working digitally with clients [3]. |
|  | Year of birth | 1958–2005 |  |  |
| *Position* | Do you work as a nurse manager? | - - - 1. No       2. Yes, in lower management       3. Yes, in middle management       4. Yes, in upper management | 1 = Staff nurse (response option 1) 2 = Nurse manager (2–4) |  |
| *Training opportunities for EHR use* | Please assess the training or orientation related to the use of information systems: Employer offers continuous training in electronic health records or client information systems use. | 1. Fully agree 2. Agree 3. Neither agree nor disagree 4. Disagree 5. Fully disagree | Of the respondents, 1531 nurses fully disagreed or disagreed the statement, 500 were neutral, and 884 agreed or fully agreed. The variable was recoded to fully understand the effect of agreement compared to other opinions as well as to simplify the interpretation of the analysis: 1 = Disagree–neutral (response options 3–5)  2 = Agree (1–2). | The same measure has previously been associated, for example, with giving a good grade to the EHR system in use [5]. |
| *Supportive HISs* | Use the following statements to assess how the information systems you use support carrying out your duties:   1. Information systems help in preventing errors and mistakes associated with medications. 2. Information systems help to avoid duplicate tests and examinations. 3. Information systems help to ensure continuity of care. 4. The electronic health record/client information system generates a summary view that helps to form an overall picture of the patient’s health status. 5. Information systems help to improve quality of care. 6. Use of information system speeds up decision-making in patient care. | 1. Fully agree 2. Agree 3. Neither agree nor disagree 4. Disagree 5. Fully disagree | The response options were reverse coded. The six items were used as a composite mean score (α = 0.85). | Similar items were first used in studies among physicians [6] and were later applied to the work of nurses [7]. In a previous nursing study, nursing competence was associated with a measure based on nurses’ perceptions of similar HIS statements [8]. |
| *Digital client work* | Does your work involve digital patient or client contact? This does not refer to work carried out by telephone. | 1. No 2. Yes | 1 = No  2 = Yes | The same question was used as a dependent variable in a previous nursing study, which discovered that the working unit and digital dedication were associated with working digitally with clients [3]. |
| *Number of HISs used in client work* | How many information systems do you log in to daily when working with clients/patients? (This refers to separate logins using a username or an ID card to systems, which are used to record client or patient data.) | 1. 0 2. 1 3. 2 4. 3 5. 4 6. 5 or more | 1 = 1–2 (including those part-time workers and nurse managers who answered ‘0’, because they used at least one system but did not log into them on a daily basis) 2 = 3 or more |  |
| **Background variables** | | | | |
| *Professional qualification* | Professional qualification in healthcare | 1. Registered nurse 2. Public health nurse 3. Midwife 4. Paramedic 5. Other | 1 = Registered nurse  2 = Public health nurse  3 = Midwife  4 = Paramedic  5 = Other nursing qualification, such as specialist qualifications or those based on earlier educational structures |  |
| *Gender* | Gender | 1. Man 2. Woman 3. Other 4. I prefer not to say | 1 = Woman (response option 2)  2 = Man (1)  3 = Other or prefer not to say (3–4) |  |
| *Workplace* | Where do you work more specifically? (If you work in several workplaces, answer the survey based on the workplace where you work the most hours.) | A list of 23 different workplaces located in hospitals, health and social care centres, and other settings | 1 = Acute care (including hospital emergency clinic, intensive care or monitoring unit, operating room, delivery room, public health centre emergency clinic, emergency care or ambulance)  2 = Inpatient care (hospital inpatient ward, public health centre inpatient ward)  3 = Outpatient care (outpatient clinic, patient/client appointment, maternity health clinic, child health clinic, occupational health, school or student healthcare)  4 = Home-based care (home nursing, hospital-at-home)  5 = Supportive housing and care facilities (service housing, supported housing, housing services, service housing with 24/7 assistance, institutional care)  6 = Other (such as other hospital unit, other health and social centre unit, private medical clinic or centre, laboratory or imaging services at research unit). |  |
| *Location of employment* | In which geographical area is your workplace located? Please select the area where the unit where you primarily work is located. | A list of 21 wellbeing service counties, the city of Helsinki, and Åland | Respondents from Åland were recoded with the nearest county on the mainland, Southwest Finland wellbeing services county. |  |
| *Digital work skills* | How well do you feel you master the following skills required by information systems? : Working in a digital healthcare environment | 1. Excellently 2. Well 3. Satisfactorily 4. Passably 5. My organisation does not require this skill | 1 = Tolerable–satisfactory (response options 3–4)  2 = Good–excellent (1–2).  Response option 5 was coded as missing (n = 10). |  |
| *Stress related to HISs* | How often has each of the issues mentioned below clearly disturbed, worried, or burdened you at work during the past 6 months?   1. Changing information systems. 2. Awkward, poorly functioning IT equipment or software. | 1. Constantly 2. Very often 3. Quite often 4. Quite rarely 5. Very rarely 6. Never | The response options were reverse coded. The two items were used as a composite mean score (α = 0.73). | The measure has previously been used in studies among nurses and physicians and has been associated with, for example, stress and psychological distress [9–11]. |

## **Additional file 1 references**

[1] Finnish Institute for Health and Welfare (THL). A 2023 survey on information systems for registered nurses; 2023. https://thl.fi/documents/155392151/0/A_2023_survey_on_information_systems_for_registered_nurses+(1).pdf/9413c80c-3726-1aae-bae4-6bbfb6d134c1/A_2023_survey_on_information_systems_for_registered_nurses+(1).pdf?t=1710244110813. Accessed 10 Jan 2025.

[2] Mäkiniemi J-P, Ahola S, Joensuu J. A Novel Construct To Measure Employees’ Technology-Related Experiences of Well-Being: Empirical Validation of the Techno-Work Engagement Scale (TechnoWES). Scand J Work Organ Psychol. 2020;5:4. https://doi.org/10.16993/sjwop.79.

[3] Kainiemi E, Kaihlanen A-M, Virtanen L, Vehko T, Heponiemi T. Registered Nurses’ Digital Client Work and Associating Factors: A Cross-Sectional Study. J Adv Nurs. 2024. https://doi.org/10.1111/jan.16485.

[4] Virtanen L, Kainiemi E, Vehko T, Heponiemi T, Kaihlanen AM. Primary care nurses’ readiness to promote digital health technology use among patients. Eur J Public Health. 2024;34:ckae144.194. https://doi.org/10.1093/eurpub/ckae144.194.

[5] Kyytsönen M, Kaihlanen A-M, Kinnunen U-M, Saranto K, Vehko T. The association of implementation time, skills, and education with registered nurses evaluation of a client information system and electronic health record. FinJeHeW. 2024;16:81–94. https://doi.org/10.23996/fjhw.137700.

[6] Viitanen J, Hyppönen H, Lääveri T, Vänskä J, Reponen J, Winblad I. National questionnaire study on clinical ICT systems proofs: Physicians suffer from poor usability. Int J Med Inform. 2011;80:708–25. https://doi.org/10.1016/j.ijmedinf.2011.06.010.

[7] Hyppönen H, Lääveri T, Hahtela N, Suutarla A, Sillanpää K, Kinnunen U-M, et al. Smart systems for capable users? Nurses’ experiences on patient information systems 2017. FinJeHeW. 2018;10:30–59. https://doi.org/10.23996/fjhw.65363.

[8] Kaihlanen A-M, Elovainio M, Virtanen L, Kinnunen U-M, Vehko T, Saranto K, et al. Nursing informatics competence profiles and perceptions of health information system usefulness among registered nurses: A latent profile analysis. J Adv Nurs. 2023;79(10):4022–4033. https://doi.org/10.1111/jan.15718.

[9] Heponiemi T, Hyppönen H, Kujala S, Aalto A-M, Vehko T, Vänskä J, et al. Predictors of physicians’ stress related to information systems: a nine-year follow-up survey study. BMC Health Serv Res. 2018;18:284. https://doi.org/10.1186/s12913-018-3094-x.

[10] Heponiemi T, Kujala S, Vainiomäki S, Vehko T, Lääveri T, Vänskä J, et al. Usability Factors Associated With Physicians’ Distress and Information System–Related Stress: Cross-Sectional Survey. JMIR Med Inform. 2019;7:e13466. https://doi.org/10.2196/13466.

[11] Kaihlanen A-M, Gluschkoff K, Laukka E, Heponiemi T. The information system stress, informatics competence and well-being of newly graduated and experienced nurses: a cross-sectional study. BMC Health Serv Res. 2021;21:1096. https://doi.org/10.1186/s12913-021-07132-6.
