# Supplementary material for: Digitally dedicated nurses: a nationwide cross-sectional study of associated career and digital factors in the workplace
Source: BMC Health Serv Res. 2025 Oct 15;25:1365. doi: 10.1186/s12913-025-13333-0 (PMC12522463; doi:10.1186/s12913-025-13333-0)
Supplement: Supplementary file 2 — Additional file 2. Regression coefficients for background variables. Linear regression coefficients for background variables in models examining digital dedication among registered nurses [file 12913_2025_13333_MOESM2_ESM.docx]

# **Additional file 2**

## **Regression coefficients for background variables**

Table A2 presents the linear regression coefficients for all background variables used as adjustments in the multivariable models examining digital dedication among registered nurses.

**Table A2**

*Linear regression coefficients for background variables in models examining digital dedication among registered nurses*

| Background variable | | Univariable models  n = 2846–2901 | | Multivariable model 1  n = 2777 | | Multivariable model 2  with an interaction term n = 2777 | |
| --- | --- | --- | --- | --- | --- | --- | --- |
|  |  | *b* | 95% CI | *b* | 95% CI | *b* | 95% CI |
| Gender | |  |  |  |  |  |  |
|  | Woman | ref. |  | ref. |  | ref. |  |
|  | Man | −0.004 | −0.158, 0.150 | −0.003 | −0.154, 0.148 | −0.006 | −0.157, 0.145 |
|  | Other or prefer not to say | −0.221 | −0.638, 0.196 | −0.186 | −0.604, 0.233 | −0.194 | −0.612, 0.225 |
| Workplace | |  |  |  |  |  |  |
|  | Acute care | ref. |  | ref. |  | ref. |  |
|  | Inpatient care | −0.062 | −0.190, 0.065 | −0.030 | −0.155, 0.094 | −0.028 | −0.152, 0.097 |
|  | Outpatient care | **0.185** | **0.064, 0.306** | 0.080 | −0.045, 0.201 | 0.081 | −0.045, 0.206 |
|  | Home-based care | 0.099 | −0.082, 0.280 | 0.030 | −0.146, 0.205 | 0.022 | −0.154, 0.197 |
|  | Supportive housing and care facilities | 0.034 | −0.121, 0.189 | −0.051 | −0.207, 0.105 | −0.051 | −0.206, 0.105 |
|  | Other | **0.163** | **0.017, 0.309** | 0.086 | −0.058, 0.230 | 0.085 | −0.580, 0.229 |
| Location of employment (wellbeing service county) | |  |  |  |  |  |  |
|  | Pirkanmaa | ref. |  | ref. |  | ref. |  |
|  | East Uusimaa | −0.047 | −0.398, 0.303 | 0.014 | −0.317, 0.344 | 0.014 | −0.316, 0.344 |
|  | Central Uusimaa | −0.031 | −0.260, 0.198 | −0.002 | −0.226, 0.221 | −0.011 | −0.234, 0.213 |
|  | West Uusimaa | 0.025 | −0.174, 0.224 | 0.065 | −0.127, 0.256 | 0.073 | −0.119, 0.265 |
|  | Vantaa and Kerava | −0.038 | −0.316, 0.240 | 0.010 | −0.261, 0.280 | 0.016 | −0.255, 0.286 |
|  | Southwest Finland and Åland | 0.086 | −0.090, 0.262 | 0.030 | −0.139, 0.198 | 0.032 | −0.136, 0.201 |
|  | Satakunta | 0.025 | −0.195, 0.245 | 0.052 | −0.159, 0.262 | 0.049 | −0.161, 0.260 |
|  | Kanta-Häme | −0.087 | −0.366, 0.191 | −0.060 | −0.324, 0.204 | −0.055 | −0.320, 0.209 |
|  | Päijät-Häme | 0.068 | −0.175, 0.310 | 0.075 | −0.158, 0.308 | 0.072 | −0.161, 0.305 |
|  | Kymenlaakso | −0.120 | −0.372, 0.134 | −0.075 | −0.317, 0.168 | −0.062 | −0.305, 0.181 |
|  | South Karelia | 0.134 | −0.142, 0.409 | 0.096 | −0.167, 0.359 | 0.099 | −0.164, 0.362 |
|  | South Savo | 0.083 | −0.181, 0.348 | 0.137 | −0.118, 0.392 | 0.150 | −0.105, 0.404 |
|  | North Savo | 0.011 | −0.199, 0.221 | 0.069 | −0.133, 0.271 | 0.067 | −0.135, 0.269 |
|  | North Karelia | −0.187 | −0.476, 0.103 | −0.134 | −0.414, 0.146 | −0.145 | −0.425, 0.136 |
|  | Central Finland | **0.280** | **0.059, 0.502** | **0.245** | **0.034, 0.456** | 0.248 | 0.037, 0.459 |
|  | South Ostrobothnia | 0.072 | −0.163, 0.306 | 0.029 | −0.196, 0.253 | 0.028 | −0.196, 0.253 |
|  | Ostrobothnia | 0.147 | −0.128, 0.422 | 0.067 | −0.199, 0.334 | 0.068 | −0.198, 0.334 |
|  | Central Ostrobothnia | −0.053 | −0.368, 0.262 | −0.026 | −0.328, 0.276 | −0.030 | −0.331, 0.272 |
|  | North Ostrobothnia | 0.156 | −0.027, 0.340 | 0.083 | −0.093, 0.259 | 0.089 | −0.087, 0.265 |
|  | Kainuu | 0.112 | −0.198, 0.422 | −0.059 | −0.355, 0.237 | −0.050 | −0.346, 0.245 |
|  | Lapland | 0.040 | −0.210, 0.290 | −0.002 | −0.243, 0.239 | −0.003 | −0.243, 0.238 |
|  | City of Helsinki | 0.145 | −0.029, 0.318 | **0.253** | **0.083, 0.423** | **0.256** | **0.086, 0.426** |
| Digital work skills | |  |  |  |  |  |  |
|  | Tolerable–Satisfactory | ref. |  | ref. |  | ref. |  |
|  | Good–Excellent | **0.325** | **0.218, 0.434** | **0.155** | **0.048, 0.261** | **0.155** | **0.048, 0.261** |
| Stress related to HISs | | **−0.159** | **−0.193, −0.126** | **−0.108** | **−0.144, −0.072** | **−0.107** | **−0.143, −0.071** |

*Note. b* Unstandardised beta coefficient indicates the average change in the digital dedication score (on a scale of 1–5) under the following conditions: a) in a specific variable group compared to a reference group (for categorical independent variables), or b) for a one-unit change in supportive HISs (a continuous independent variable). *CI* Confidence interval, *ref.* Reference, *HISs* Health information systems. The two multivariable models include all independent variables (career stage, career position, training opportunities for electronic health record use, supportive HISs, number of HISs used in client work, and digital client work) reported in Table 2 in the main text, along with the listed background variables used for adjustment. Multivariable model 2 also includes the interaction term ‘Career stage × Training opportunities for electronic health record use’. Statistically significant values (*p* < .05) are highlighted in bold.
